# Guy's Hospital

**Published:** 1830-09

**Authors:** 


### GUY'S HOSPITAL.

### *Case of Aneurism of the Arteria Innominata; Operation of tying the Carotid. Sudden Death; singular Pathological Appearances.*

Elizabeth Goodman, æt. sixty-one, observed, in the month of September, 1828, a pulsating tumor immediately above the right side of her sternum. This progressively enlarged till her admission into Guy's Hospital, about October 15th, 1828, when it was as large as an egg, and rather flattened. The following note of the appearances was made at that time: "The tumor rises from behind the right sterno-clavicular articulation, ascends to near one third of the length of the sterno-mastoideus muscle, and extends outwards about one third along the clavicle. Its pulsation is synchronous with the pulse, and then dilates on all sides, though pretty firm. The tumor has lately been very painful; numbness extends up the neck and along the right arm; nor can she lie comfortably on the right side.

"She is a swarthy little woman, of spare habit and regular living; has had a family, and worked hard. Her pulse is sixty in the minute, hard and full on the right side, weaker on the left."

An occasional aperient was given her, and a belladonna plaster applied to the tumor.

In the course of six weeks, the tumor appeared somewhat larger, though she did not suffer so much pain from the pressure on the axillary and cervical nerves. Her pulse rose to ninety, and the difference in fulness between the pulse of the two arms became more apparent. She did not suffer much dyspnœa.—V.S. to ten ounces. Capiat Træ. Digit. m. x. ex Liq. Ammon. Acetat. bis die. Rep. Empl. Bellad. p. r. n.

Under the occasional use of these measures, she improved till the following spring, when she left the hospital sufficiently well, as she thought, to work for her livelihood. The tumor was now stationary; for neither the painful numbness, nor any dyspnœa, nor the external enlargement, had increased.

In March 1830, she was again admitted by Mr. Key, on account of aggravation of all her symptoms, especially her difficulty of breathing on going up stairs, and the painful numbness of the right arm being now so great that she could not sleep. During her absence her health has been pretty good, and she has undergone much exertion. The tumor has not enlarged so much as might have been expected: though somewhat dilated on all sides, it is altered chiefly by the sensation of the sac being thinned; its pulsation is very violent, and it is very tender.—Capiat Opii gr. ss. o. n. Empl. Bellad. tumori. Sumat Træ. Digit. m. x. ex Liquor Salin. t. d.

Her symptoms were again palliated, though the weakness and faintness induced by the digitalis obliged it to be omitted.

In April the aneurism enlarged forwards very much, in some degree dislocating the right sterno-clavicular articulation. It was therefore rendered much more prominent, ascending behind the sterno-mastoid muscle (which, with the jugular vein, was pushed a little outwards,) to the distance of two inches above the clavicle, and extending outwards to nearly three inches along that bone. Of course, its pulsation could be felt far beyond these points.

As the health of the woman was good, as she was very desirous that some curative means should be adopted, and as her difficulty of breathing was much diminished, Mr. Key determined, as the only chance of averting otherwise sudden death, to tie the carotid artery, and, if necessary, the subclavian also at a subsequent period. In this resolution he was borne out by the corroborative opinions of Sir A. Cooper, Professor Ekstrom, of Stockholm, Professor Galenzouski, of Wilna, and others. These gentlemen appeared to consider that the aneurism was confined to the arteria innominata, and could discover no indication of the heart or aorta being otherwise than healthy.

*Operation.* The operation was performed at half-past one P.M. on Tuesday, the 20th July. The patient walked into the theatre and was laid on pillows on a high table, in a supine posture; her head and shoulders raised, and the former lying on its left side, so as to stretch the soft parts of the neck somewhat. Mr. Key stood on the right side of the patient, and proceeded to operate as follows:

Commencing at the corner of the os hyoides, an incision was made downwards for an inch and a half along the anterior belly of the sterno-cleido-mastoideus, (this being the extent of the space between the tumor and the os hyoides), so as to lay bare the cervical fascia and platysma myoides. Several little veins were found to run across the line of incision, all of which, except two, were avoided; then, by gradual steps, Mr. K. proceeded, using the edge of the scalpel cautiously, till he exposed the upper edge of the omo-hyoideus muscle: here he laid aside the knife, and, by means of a director, separated the cellular membrane down to the sheath of the artery, on which was running a branch of the descendens noni nerve. Having laid bare the vessel, which appeared deep seated, he passed an aneurismal needle, armed with a silk ligature, under it, from within outwards. With the usual precautions, the ligature was made fast, the coats of the artery appearing very readily to give way. The whole operation occupied fifteen minutes, and not more than half an ounce of blood was lost. The patient bore it without a murmur, and complained of pain only when the vessel was tied.

At the instant of the ligature being tightened, the tumor first fluttered in its beat, and then became decidedly smaller, both in bulk and in its force of pulsation. The artery at the right wrist was not in the least affected, nor did the patient herself feel faint or sick, or in any way incommoded by the operation; for she spoke cheerfully, and appeared as well as before its performance. This diminution in the bulk of the tumor, however, was not permanent, for, before she had left the theatre, it was nearly as large as previous to the operation, though its beat was certainly fainter.

*State of the patient after the operation.* She was immediately carried to bed, and appeared cheerful; her pulse ninety, rather sharp, but also irregular in its beat; the tumor as before the operation in bulk, but with a fainter and more irregular beat; her head was raised on a high pillow, and laid on the left side. She had not been in bed more than half an hour before she suddenly raised herself, gasped for breath, and called for something to drink; she then fell into a severe fit of coughing, which lasted, with little remission, for several minutes: her distress was such as to create an apprehension of instant dissolution from the rupture of the sac. She was, however, able to take a little ammonia, with ten drops of laudanum, which succeeded in quieting her immediately. Her back was supported by high pillows, and she was again quite calm. In about an hour and a half from the operation, she appeared to be asleep; her breathing was natural, attended only by a peculiar snore, which did not now attract notice, because it was habitual to her: this noise gradually became fainter, till at length it quite died away. She was thought to be in a comfortable sleep, and therefore not disturbed till about half-past five o'clock, when, on being seen, it was found that there was no pulse in the wrists, and that the heart was beating with a scarcely perceptible flutter. It was not possible to afford any relief, for in a few seconds more she died. From the time of the paroxysm of coughing she had not spoken, but appeared perfectly quiet, and died in the most calm, tranquil, and unconscious manner possible.

The body was examined twenty hours afterwards. Both pleuræ were natural in appearance, but contained between them about a pint and a half of serous fluid. There was about four ounces of serum in the pericardium; there was no extravasation of blood any where; the sac was adherent to the upper part of the sternum, and adjacent part of the clavicle and first rib. These, together with the heart, left lung, and the soft parts in the neck, as far as the lower jaw, were now removed together, and a careful examination made.

The whole arch of the aorta, from the heart to its termination in the thoracic portion, was very much dilated, and all its inner surface quite rough, with numerous ossific scales spread on it: the heart itself was sound, and the semilunar valves nearly natural. Besides this, there arose a distinct sac from the right wall of the arteria innominata, and from the adjoining part of the arch, which was about the size of a small orange. This tumor had risen up on the neck, had pressed forwards the sterno-clavicular joint, had passed along the subclavian artery, and in less degree along the carotid, constituting the swelling which had been felt externally during life. This sac was more than half filled with laminæ of fibrin, in various degrees of consolidation. The carotid and subclavian arteries were both quite sound, and not at all involved in the disease. The part of the innominata near the bifurcation was also lined with ossific scales, like the aorta, and its opening much enlarged. The ligature was fairly on the carotid, about two inches below its division.

Upon prosecuting the dissection of the parts with a view to preparation, a most unusual circumstance was discovered, viz. the left carotid opened from the arch of the aorta by an orifice scarcely large enough to admit a small probe. It appeared as if a membrane had formed across its original orifice, very nearly resembling the membrane over the foramen ovale in the septum of the auricles of the heart; but what is still more singular is, that the carotid itself, from the aorta to the bifurcation, retained its natural caliber, though supplied entirely through a mouth scarcely one tenth part of its natural size. There was no vessel whatever entering any part of this common carotid which could in any way have conveyed blood to it. At its division, the external carotid continued of its natural size, but the internal became abruptly very small, almost immediately after its commencement. The interior of this, the left common carotid, contained an adherent layer of fibrin similar to that in the aneurismal sac, and not at all like the strings of fibrin ordinarily found in blood-vessels. This appeared to be a step towards the gradual obliteration of the artery.

The subclavian was of its usual size, and the vertebrals were rather small than otherwise on both sides; so that, when the right carotid was tied, the brain was so very much deprived of its supply of blood as to render it unable to support the actions of life; and hence the quick and perfectly quiet dissolution which followed.

The brain was examined. It was found healthy; its vessels were quite sound, and contained the ordinary quantity of blood. There was a little serous effusion between the membranes.

The abdominal viscera were generally sound, except a small polypus in utero, which grew from just below the orifice of the left fallopian tube. The descending aorta was quite natural.*

* Medical Gazette.

